# Outcome of haematology patients admitted to intensive care in a tertiary centre: primary haematological diagnosis, recent chemotherapy and bone marrow transplantation are not associated with outcome

**DOI:** 10.1186/cc9917

**Published:** 2011-03-11

**Authors:** R José, I McDonald, P Pfeffer, S Shaw, B Agarwal

**Affiliations:** 1 Royal Free Hospital, London, UK

## Introduction

Acute illness in patients with underlying haematological disease is associated with poor prognosis. Recent reports suggest improved outcomes in these patients except for those with bone marrow transplantation (BMT) or recent chemotherapy [1]. We sought to audit the overall outcome and potential determinants of outcome in these patients admitted to our ICU.

## Methods

Retrospective data on demographics, underlying haematological diagnosis, BMT, recent chemotherapy, reason for acute illness, severity of acute physiological derangement (severe acute physiological score (SAPS), number of failed organs, need for invasive mechanical ventilation and renal support), and ICU and hospital outcomes were collected for 106 consecutive admissions (97 patients) between January 2005 and December 2008. Re-admissions were excluded (9/106 patients). Data were analysed with SPSS software.

## Results

Of the 97 patients, NHL (30.9%) and AML (26.8%) accounted for most haematological diagnoses. A total 24.7% were post-BMT, and 36.1% had chemotherapy within a month of admission or on the ICU. The mean (SD) age was 49 (15), SAPS 55 (16) and 56.7% were males. The mortality at ICU and hospital discharge was 51.5% and 63.9%, respectively. Gender, neutropaenia (≤1 × 10^9^/l), haematological diagnosis, admission reason, HIV status, BMT and recent chemotherapy were not predictive of ICU or hospital outcome (*P *> 0.05). SAPS, invasive mechanical ventilation (IMV), renal support (RS) and sequential number of organs supported (OS) were predictive of both ICU and hospital mortality outcomes (*P *< 0.05).

## Conclusions

There is ongoing heightened risk of mortality in patients with haematology diagnoses admitted to the ICU with acute illness, related to both the severity of the initial physiological disturbance and requirements for organ support. In our patient population, BMT and recent chemotherapy were not associated with increased mortality, but this will need further evaluation with a larger sample size.
